# Increasing skeletal muscle carnitine content in older individuals increases whole‐body fat oxidation during moderate‐intensity exercise

**DOI:** 10.1111/acel.13303

**Published:** 2021-01-19

**Authors:** Carolyn Chee, Chris E. Shannon, Aisling Burns, Anna L. Selby, Daniel Wilkinson, Kenneth Smith, Paul L. Greenhaff, Francis B. Stephens

**Affiliations:** ^1^ MRC/Versus Arthritis Centre for Musculoskeletal Ageing Research School of Life Sciences University of Nottingham Nottingham UK; ^2^ Diabetes Division University of Texas Health Science Centre San Antonio TX USA; ^3^ Sam and Ann Barshop Institute for Longevity and Aging Studies University of Texas Health Science Centre San Antonio TX USA; ^4^ MRC/Versus Arthritis Centre for Musculoskeletal Ageing Research School of Medicine University of Nottingham Nottingham UK; ^5^ Department of Sport and Health Sciences St Luke's Campus University of Exeter Exeter UK

**Keywords:** carnitine, fat oxidation, insulin resistance, intramyocellular lipid, older adults, skeletal muscle

## Abstract

Intramyocellular lipid (IMCL) utilization is impaired in older individuals, and IMCL accumulation is associated with insulin resistance. We hypothesized that increasing muscle total carnitine content in older men would increase fat oxidation and IMCL utilization during exercise, and improve insulin sensitivity. Fourteen healthy older men (69 ± 1 year, BMI 26.5 ± 0.8 kg/m^2^) performed 1 h of cycling at 50% VO_2_max and, on a separate occasion, underwent a 60 mU/m^2^/min euglycaemic hyperinsulinaemic clamp before and after 25 weeks of daily ingestion of a 220 ml insulinogenic beverage (44.4 g carbohydrate, 13.8 g protein) containing 4.5 g placebo (*n* = 7) or L‐carnitine L‐tartrate (*n* = 7). During supplementation, participants performed twice‐weekly cycling for 1 h at 50% VO_2_max. Placebo ingestion had no effect on muscle carnitine content or total fat oxidation during exercise at 50% VO_2_max. L‐carnitine supplementation resulted in a 20% increase in muscle total carnitine content (20.1 ± 1.2 to 23.9 ± 1.7 mmol/kg/dm; *p* < 0.01) and a 20% increase in total fat oxidation (181.1 ± 15.0 to 220.4 ± 19.6 J/kg lbm/min; *p* < 0.01), predominantly due to increased IMCL utilization. These changes were associated with increased expression of genes involved in fat metabolism (ACAT1, DGKD & PLIN2; *p* < 0.05). There was no change in resting insulin‐stimulated whole‐body or skeletal muscle glucose disposal after supplementation. This is the first study to demonstrate that a carnitine‐mediated increase in fat oxidation is achievable in older individuals. This warrants further investigation given reduced lipid turnover is associated with poor metabolic health in older adults.

## Graphical abstract

High Intramyocellular lipid (IMCL) content in older individuals, likely due to reduced fat oxidation, is associated with insulin resistance. The present study demonstrated that increasing muscle carnitine content in healthy older males resulted in a 20% increase in fat oxidation during exercise, likely due to an increase in IMCL utilization. This was supported by changes in expression of genes associated with IMCL turnover and oxidation, but no improvement in insulin sensitivity was observed.

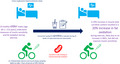

## 1 INTRODUCTION

Intramyocellular lipid (IMCL) tends to be greater in older individuals, particularly in subsarcolemmal (SSL) regions (Chee et al., 2016; Crane et al., 2010), and has been strongly associated with insulin resistance (Chee et al., 2016; Li et al., 2014; Nielsen et al., 2010; Pan et al., 1997; Perseghin et al., 1999; Petersen et al., 2003). Why IMCL accumulates with increasing age is not clear, but several studies have demonstrated reduced free fatty acid oxidation in older individuals, perhaps due to reduced mitochondrial function (Petersen et al., 2003), despite increased whole‐body lipolysis and fatty acid availability compared to young at rest and during exercise (Sial et al., 1996; Solomon et al., 2008). Indeed, using an intravenous [U‐^13^C]palmitate infusion approach, we (Chee et al., 2016) and others (Boon et al., 2007) have demonstrated that the elevated rate of appearance of plasma fatty acids in response to exercise at 50% maximal oxygen consumption (VO_2_max) is associated with a reduction in the relative contribution of IMCL to total fat oxidation in older individuals. However, a novel finding from this work (Chee et al., 2016) was that lean older men, who had comparable whole‐body and skeletal muscle insulin sensitivity and SSL IMCL content to lean young men, were able to oxidize a larger proportion of the excess plasma‐free fatty acids during exercise compared to overweight older men, in whom SSL lipid content increased during exercise. Indeed, lean older men had a greater mRNA expression (Chee et al., 2016) and maximal activity (Coggan et al., 1985) of β‐hydroxyacyl‐CoA dehydrogenase, an intramitochondrial enzyme that catalyses a rate‐limiting step in β‐oxidation, than younger counterparts of similar habitual physical activity levels. Moreover, exercise training has been shown to improve basal fat oxidation and prevent IMCL accumulation in older individuals (Sial et al., 1996; Solomon et al., 2008), and an improvement in insulin sensitivity has been previously observed with reduced SSL IMCL following several months of exercise training where the capacity to oxidize fatty acids was increased (Li et al., 2014; Nielsen et al., 2010). Thus, it would appear that a relatively high rate of mitochondrial fatty acid flux is integral to preventing the IMCL accumulation and insulin resistance often associated with age, but this has not been tested experimentally.

Free carnitine plays an essential role in regulating fatty acid flux by facilitating the translocation of long‐chain fatty acids into mitochondria for subsequent β‐oxidation via the rate‐limiting carnitine palmitoyltransferase 1 (CPT1) reaction (Fritz & McEwen, 1959). We have previously demonstrated that twice daily ingestion of 1.36 g L‐carnitine in combination with a beverage containing 80 g of carbohydrate (in order to stimulate insulin‐mediated muscle carnitine accumulation) for up to 24 weeks can increase skeletal muscle total carnitine content by around 20% in young healthy men (Stephens et al., 2013; Wall et al., 2011). This resulted in an 80% increase in muscle‐free carnitine availability during exercise at 50% VO_2_max, which was associated with a 6% increase in energy expenditure. The increase in energy expenditure was likely due to a carnitine‐mediated increase in fatty acid oxidation, which corresponded with a 50% reduction in muscle glycogen utilization during exercise (Stephens et al., 2013; Wall et al., 2011). Moreover, this manipulation of skeletal muscle fuel metabolism prevented the increase in body fat mass associated with daily ingestion of a high carbohydrate beverage, and resulted in an adaptive increase in the expression of gene networks involved in insulin signalling, peroxisome proliferator‐activated receptor (PPAR) signalling, and fatty acid metabolism (Stephens et al., 2013). Thus, it would appear that muscle‐free carnitine availability is limiting to the CPT1 reaction and the rate of fatty acid oxidation *in vivo* at moderate, as well as high exercise intensities (van Loon et al., 2001; Petrick & Holloway, 2019). Accordingly, increasing muscle carnitine content is an ideal approach to further investigate the role of mitochondrial fatty acid flux and IMCL turnover in older individuals. Therefore, the aim of the present study was to test the hypothesis that a dietary mediated increase in skeletal muscle carnitine content would increase the rate of plasma fatty acid oxidation during moderate‐intensity exercise in older male participants. We also aimed to assess whether any effect would be of a magnitude to reduce resting IMCL content, insulin resistance and fat mass over 25 weeks of moderate‐intensity exercise prescription in older individuals.

## 2 METHODS

### 2.1 Ethical approval and exercise testing

Based on an anticipated 20 ± 15% increase in total fatty acid oxidation following 24 weeks of skeletal muscle carnitine loading (we have previously observed a 10% increase in fat oxidation following 12 weeks, Stephens et al., 2013), an alpha of 0.05, and a power of 0.80, we required 20 participants to be recruited to allow for a 20% drop out rate. Twenty participants were recruited but 6 dropped out at various stages for personal reasons or problems with data collection. We did not perform intention to treat analysis. Thus, 14 healthy, non‐smoking, male volunteers (age 69.1 ± 0.6 years; body mass index (BMI) 26.5 ± 0.8 kg/m^2^) were included in data analysis for the present study. The study was approved by the University of Nottingham's Medical School Ethics Committee in accordance with the Declaration of Helsinki. Written informed consent was obtained from all participants before taking part in the study, and they were made aware that they were free to withdraw at any point. All volunteers underwent routine medical screening and completed a general health questionnaire indicating their habitual frequency of performing physical activities including resistance exercise, running, cycling and swimming. Exclusion criteria included evidence of metabolic disease, cardiovascular disease, overt muscle wasting, cerebrovascular disease, respiratory disease, inflammatory bowel disease, renal disease or a clotting dysfunction. Participants prescribed statins, aspirin or NSAID’s were instructed to continue medication. On a separate visit, all participants performed a continuous incremental exhaustive exercise test on an electronic‐braked cycle ergometer (Excalibur, Lode, The Netherlands) to determine their maximal rate of oxygen consumption (VO_2_max; Quark CPET, Cosmed, Italy). To calculate the workload that would elicit 50% VO_2_max, the test started at a workload of 1 W/kg body mass and involved four 3 min stages of 20 to 30 W increments, followed by several 1 min stages of 20 to 30 W increments to exhaustion in order to minimize the duration of strenuous exercise and the risk of adverse cardiovascular events. Heart rate was measured throughout. The workload corresponding to 50 ± 2.5% VO_2_max was confirmed in a familiarization visit at least 3 days later.

### 2.2 Experimental protocol

Subjects reported to the David Greenfield Human Physiology Unit at the University of Nottingham Medical School on three occasions. On the first occasion, they completed a quality of life (SF‐36) questionnaire and performed an incremental shuttle walking test (ISWT). Briefly, the test required the patients to walk up and down a 10‐metre course, set out in a quiet corridor of the Unit, at a gradually increasing speed, paced by audio signals (bleeps), until they were exhausted and/or could no longer keep up with the bleeps, with total distance walked taken as a measure of functional exercise capacity (Singh et al., 1992). The exercise test was originally designed to provoke a symptom‐limited maximal performance in chronic obstructive pulmonary disease patients in order to provide objective measurement of disability, but has also been used to assess functional exercise capacity in older individuals (Harrison et al., 2013).

On the second visit (*Resting* visit), they arrived at the unit at approximately 0800 after an overnight fast, having abstained from exercise and alcohol for the previous 48 h. On arrival, lean soft tissue mass (kg), fat mass (kg) and bone mineral content (kg) of standard body regions were measured using dual‐energy X‐ray absorptiometry (DXA; Lunar Prodigy, GE Healthcare, US). Participants were then asked to rest in a supine position on a bed while cannulae were inserted into a vein on the dorsal surface of the hand for arterialized‐venous blood sampling (Gallen & MacDonald, 1990), the forearm for the infusion of insulin (Actrapid; Novo Nordisk, Denmark) and 20% dextrose (Baxter Healthcare, UK), and the contralateral forearm for the infusion of 2‐deoxy‐D‐glucose (2DG; Sigma‐Aldrich, UK). Thereafter, a 3 h euglycaemic (4.5 mmol/l) hyperinsulinaemic (60 mU/m^2^/min) clamp was commenced in combination with the intravenous infusion of 2DG (6 mg/kg/h) to assess whole‐body and skeletal muscle insulin sensitivity, respectively, as described previously (Chee et al., 2016). VO_2_ and VCO_2_ were measured over a 20 min period of quiet rest via a ventilated hood connected to an indirect calorimeter (GEMNutrition Ltd, UK) prior to and after 2 h of the clamp. Arterialized‐venous blood was obtained every 5 min for immediate measurement of blood glucose concentrations (Stat Analyzer, YSI Inc, USA), and every 30 min for subsequent analysis of serum insulin and plasma 2DG. In addition, needle biopsy samples were obtained from the vastus lateralis (Bergstrom, 1979) before and immediately after the clamp and snap frozen in liquid nitrogen. After 3 h, the insulin and 2DG infusions were stopped, whereas the dextrose infusion was continued for approximately 80 min and the subjects were fed a high carbohydrate meal in order to stabilize blood glucose concentration. Thereafter, participants were free to leave the laboratory.

On the third visit (*Exercise* visit), at least 1 week later, volunteers again reported to the Unit at approximately 0800 following an overnight fast and rested semi‐supine on a bed while cannulae were inserted into a heated hand vein for arterialized‐venous blood sampling and a forearm vein for the infusion of NaH^13^CO_3_ (Cambridge Isotope Laboratories, USA) and [U‐^13^C]palmitate (99% enriched; Cambridge Isotope Laboratories, USA) bound to 4.5% human serum albumin (Zenalb 4.5, Bio Products Laboratory Limited, UK) at a ratio of approximately 3:1 (1.94:0.64 μmol/L). Following a 63.75 μg/kg bolus of NaH^13^CO_3_ to prime the bicarbonate pool, [U‐^13^C]palmitate was infused at a rate of 0.19 mg/kg/h for 2 h, which then increased to 0.28 mg/kg/h at the onset of 1 h cycling exercise at 50% VO_2_max, again as previously described (Chee et al., 2016). Blood samples were obtained before and every 10 min during exercise, analysed immediately for blood lactate concentration (2300 Stat Analyzer; YSI Inc, USA) and, following centrifugation, plasma samples treated with tetrahydrolipostatin (30 μg/ml plasma) and EGTA/glutathione for later analysis of fatty acids and catecholamines, respectively, were stored at −80°C. Breath samples were also collected every 10 min during exercise via one‐way valve bags and introduced into vacuumed glass tubes (Exetainer, Labco Ltd, UK) for subsequent^13^CO_2_ enrichment analysis. During the last 10 min of exercise, when the^13^CO_2_ production was at a steady‐state, indirect calorimetry was performed (Quark CPET system, Cosmed, Italy). In addition, a vastus lateralis needle biopsy was obtained immediately before and after the exercise bout and processed within 10 s to minimize *ex vivo* changes in intracellular metabolism and contamination of the IMCL pool by extracellular adipocytes. A 5 mg portion was buffered in ice‐cold 3% gluteraldehyde/0.1 M sodium cacodylate (pH 7.4) and stored at 4^◦^C for subsequent electron microscopy processing, and the remainder immediately frozen in liquid nitrogen. After the exercise bout the [U‐^13^C]palmitate infusion was stopped, participants were fed lunch, and they were free to leave the laboratory.

Following the *Exercise* experimental visit, the participants were randomized in a double‐blind fashion to receive 4.5 g of either maltodextrin placebo (Control) or L‐carnitine L‐tartrate (Carnitine), which equated to 3 g of L‐carnitine (Carnipure; Lonza Group Ltd., Basel, Switzerland), once a day for 25 weeks. The participants were instructed to ingest the supplement with a 220 ml insulinogenic beverage containing 44.4 g of carbohydrate and 13.8 g of protein (Ensure Plus, Abbott Nutrition, USA) between breakfast and lunch instead of their usual mid‐morning snack. We used this alternative insulinogenic beverage because our previously used protocol of twice daily feeding of 80 g of carbohydrate could be detrimental to metabolic health and liver function in older individuals. During this 25‐week dietary intervention, period participants attended the Unit twice a week to undergo 1 h of supervised exercise on the cycle ergometer at their pre‐determined 50% VO_2_max in order to increase demand for fat oxidation and enhance any effect of increasing muscle carnitine content. The sessions were limited to twice a week and exercise workload did not change over the intervention period in order to make the intervention more easily translatable to every‐day life and to avoid any excessive training effect, which is also known to improve fat oxidation, IMCL content and insulin sensitivity. The *Resting* and *Exercise* experimental visits were then repeated after 24 and 25 weeks, respectively, with the shuttle walk test and SF36 questionnaire performed again a few days after the cessation of supplementation.

### 2.3 Sample analysis

Serum samples from the *Resting* visits were analysed for insulin using a solid‐phase^125^I radioimmunoassay kit (Human Insulin Assay, Merck Millipore, USA), and plasma samples were analysed for 2DG via gas chromatography–mass spectrometry (GC‐MS; MD800, Fisons, UK). A 30 mg portion of wet muscle tissue was homogenized for analysis of 2‐deoxy‐D‐glucose‐6‐phosphate (2DG6P) content using a commercial spectrophotometric kit method (Cosmo Bio Ltd, Japan; Saito et al., 2011). In addition, total RNA was extracted from approximately 20 mg of wet muscle tissue (Trizol reagent; Invitrogen Ltd, UK) and following generation of first‐strand cDNA (SuperScript III kit; Invitrogen Ltd, UK), the relative abundance of mRNA of 12 genes from pathways involved in FFA oxidation and IMCL metabolism was determined using custom‐designed low‐density RT‐PCR array microfluidic cards (Applied Biosystems, Foster City, CA, USA) in combination with the ABI PRISM 7900 T sequence detection system and SDS 2.1 software (Applied Biosystems, Foster City, CA, USA; Stephens et al., 2013). The threshold cycle C_T_ was automatically given by the SDS software RQ manager, and relative mRNA abundance was calculated using the ΔΔC_T_ method with each subjects’ baseline sample (0 week) as their own calibrator and α‐actin as the endogenous control. C_T_ values for α‐actin did not change across time points (data not shown).

Plasma from the *Exercise* experimental visits was analysed for total fatty acids (NEFA C kit, WAKO Chemicals, Germany) on an automated analyzer (ABX Pentra 400, Horiba Medical Ltd., France), and [U‐^13^C]palmitate and palmitate by TSQ triple quadrupole gas chromatography–mass spectrometry/mass spectrometry (GC‐MS/MS, Thermo, UK) and GC‐MS (MD800, Fisons, UK) respectively, after addition of a heptadecanoic internal standard and derivatization to their methyl esters (Husek et al., 2002). Breath^13^CO_2_ enrichment was analysed by continuous‐flow isotope‐ratio MS (CF‐IRMS; AP2003 Breath Gas System, Analytical Precision, UK; Scrimgeour et al., 1988). High‐performance liquid‐chromatography (HPLC) with electrochemical detection was used to measure plasma adrenaline and noradrenaline concentrations (MacDonald & Lake, 1985). Muscle samples for transmission electron microscopy were fixed in 1% osmium tetroxide, dehydrated in graded ethanol series and embedded in two resin blocks. Three ultrathin 70–90 nanometre sections were cut from each block, mounted on copper grids, and stained in uranyl acetate and lead acetate, with one section randomly selected to be visualized at ×4200 magnification. Approximately 40 fields of view from up to 40 longitudinal fibres were systematically randomly selected by a blinded operator using the corners of copper grid squares as a guide. This method obtained at least 6 images per sample containing a SSL region, which was required for reproducible estimation of IMCL droplet (LD) characteristics. Images were analysed using Image J to determine percentage of intermyofibrillar (IMF) and SSL area covered by LD, LD size and total number of LD per square micrometre of local tissue area, which have been previously shown (Crane et al., 2010) to produce values similar to 3D stereology volume estimates (Howald et al., 1985). In addition, a 50 mg portion muscle was freeze‐dried, dissected free of visible blood and connective tissue, pulverized and used for the quantification of free‐, acetyl‐ and long‐chain acylcarnitine content (Cederblad et al., 1990; Stephens et al., 2006), as well as glycogen, lactate and phosphocreatine (PCr) as previously described (Harris et al., 1974). Total carnitine content was calculated from the sum of free and acylcarnitine moieties.

### 2.4 Calculations and statistical analysis

A single operator analysed all DXA scans to determine leg, arm, and trunk composition using the standardized regions specified by the manufacturer (enCORE 2005 version 9.1, GE Medical Systems, Bucks, U.K.). Homeostatic model assessment of insulin resistance (HOMA‐IR) was calculated by the equation of Matthews et al. (1985) using fasting glucose (G; mmol/L) and insulin (I; mU/L) concentrations ((G × I)/22.5). Insulin sensitivity index (SI_Clamp_) was calculated using the equation of Matsuda and DeFronzo (SI_Clamp_ = M/(G × ΔI); Matsuda & DeFronzo, 1999) where steady‐state (120–180 min) glucose disposal (M) is normalized for steady‐state blood glucose concentration (G; mmol/L) and the difference between fasting and steady‐state plasma insulin concentrations (ΔI; mU/L). Indirect calorimetry calculations both at rest and during exercise were performed according to non‐protein stoichiometric equations (Frayn, 1983) and normalized to lean body mass (DXA). Total energy expenditure during exercise was calculated as the sum of energy production from fat and carbohydrate, assuming that the oxidation 1 g of triacylglycerol (862 g/mol) liberates 39.4 kJ and 1 g of glucose (180 g/mol) liberates 15.6 kJ. The rate of appearance (Ra), disappearance (Rd), and oxidation of palmitate during the final 10 min of exercise were used to calculate total plasma fatty acid kinetics by dividing the fractional contribution of plasma palmitate to total plasma fatty acid concentration as previously described (Chee et al., 2016; van Loon et al., 2001). The contribution of other fat sources was calculated by subtracting plasma fatty acid oxidation from total fat oxidation calculated via indirect calorimetry.

Differences within and between groups before and after supplementation (time and treatment effects) were analysed using a two‐way ANOVA, and differences within and between groups pre‐ and post‐exercise or euglycaemic hyperinsulinaemic clamp before and after supplementation (exercise or insulin, time and treatment effects) were analysed using a three‐way ANOVA (GraphPad Prism 7, GraphPad Software Inc, USA). When a significant interaction effect (time × treatment) was observed following two‐way ANOVA, Sidak's post hoc test was performed to identify individual differences. When a significant interaction effect (time × treatment × exercise or insulin) was observed following three‐way ANOVA, a two‐way ANOVA with Sidak's post hoc test was performed on the post‐supplementation data or, in the case of only a two‐factor interaction, the non‐significant factor data were consolidated in a two‐way ANOVA with Sidak's post hoc test. All other significant main effects from the two‐ and three‐way ANOVAs have been reported in the text but not discussed. Statistical significance was set at *p* < 0.05, and all values are presented as means ± SD.

## 3 RESULTS

### 3.1 Subject characteristics

Subject characteristics are presented in Table 1. Age, statin use, body composition, VO_2_max, functional capacity as measured by ISWT, and quality of life score as measured by SF36 questionnaire were similar between Control and Carnitine groups at baseline and following 25 weeks of supplementation. There was a time × treatment interaction for fasting blood glucose (*p* = 0.0455) and a trend for fasting blood glucose to be around 6% lower in Carnitine after 25 weeks (*p* = 0.0957). However, there was no time × treatment interaction for serum insulin concentration (*p* = 0.2345), and, as such, HOMA IR was not different between Carnitine and Control over time (time × treatment interaction *p* = 0.1431).

**TABLE 1:** Subject characteristics

	Control		Carnitine	
	0 weeks	25 weeks	0 weeks	25 weeks
Age (years)	68 ± 3	69 ± 3***	70 ± 2	70 ± 2***
Statin use (*n*)	3	3	3	3
Body mass (kg)	77.5 ± 10.4	79.9 ± 7.3	78.5 ± 10.4	78.0 ± 9.4
BMI (kg/m^2^)	25.7 ± 3.2	26.5 ± 2.6	27.2 ± 3.1	27.1 ± 3.1
Lean body mass (kg)	49.4 ± 3.7	49.3 ± 2.3	51.3 ± 7.0	50.6 ± 5.3
Fasting blood glucose (mmol/L)**	4.7 ± 0.3	4.8 ± 0.6	5.0 ± 0.3	4.7 ± 0.4
Fasting serum insulin (mU/L)	10.6 ± 4.2	12.0 ± 7.9	9.5 ± 5.0	7.9 ± 4.1
HOMA IR	2.2 ± 0.9	2.7 ± 1.9	2.2 ± 1.2	1.7 ± 1.0
VO_2_max (ml/kg/lbm/min)	41.2 ± 5.0	ND	41.9 ± 5.0	ND
ISWT (metres)	558.6 ± 96.0	555.7 ± 89.6	562.9 ± 73.9	588.6 ± 47.4
SF36v2™score (%)	89.0 ± 9.4	89.6 ± 9.5	86.0 ± 9.0	87.7 ± 7.6

All values (*n* = 7) are means ± standard deviation (SD).

ND, not determined.

*** *p* < 0.001, 25 weeks significantly greater than 0 weeks.

** *p* < 0.05, significant time × treatment interaction.

### 3.2 Skeletal muscle total carnitine content

**FIGURE 1:**
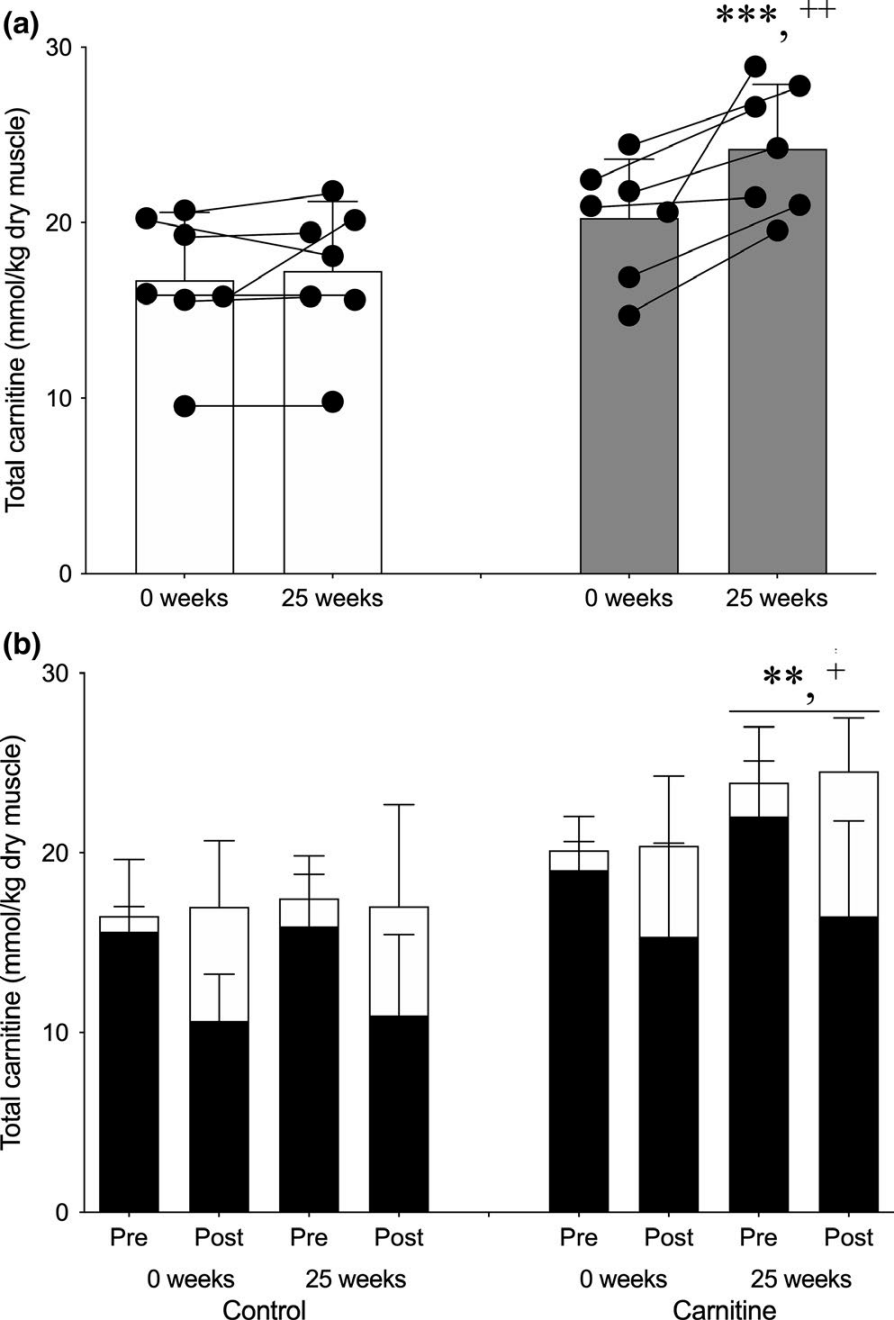
Average skeletal muscle total carnitine content (a), and the contribution of free carnitine (filled bars) and acetylcarnitine (open bars) to skeletal muscle carnitine content before (Pre) and after (Post) 1 h of exercise at 50% VO_2_max (b), before (0 weeks) and after 25 weeks of daily supplementation with 4.5 g of placebo (Control) or L‐carnitine L‐tartrate (Carnitine) in combination with 220 ml of a nutritional beverage containing 13.8 g protein and 44.4 g carbohydrate. Values represent mean ±SEM (*n* = 7). **p < 0.01, ***p < 0.001, total carnitine at 25 weeks in Carnitine significantly different to corresponding 0 weeks value. ^+^ *p* < 0.05, ^++^ *p* < 0.01, total carnitine at 25 weeks in Carnitine significantly different to corresponding Control value. Individual responses for average total muscle carnitine content (a) are represented by filled circles and connected with lines

There was a time x treatment interaction for skeletal muscle total carnitine content (*p* < 0.05). Twenty‐five weeks of supplementation had no effect on skeletal muscle total carnitine content in Control (16.7 ± 3.9 to 17.2 ± 4.0 mmol/kg dry muscle (dm); Figure 1a). In contrast, from a similar baseline value to Control, skeletal muscle total carnitine content increased by around 20% in Carnitine after 25 weeks (20.3 ± 3.4 to 24.2 ± 3.7 mmol/kg dm; *p* < 0.001; Figure 1a), such that it was also greater than Control (*p* < 0.01; Figure 1a). There was no effect of exercise on skeletal muscle total carnitine content in either group over the twenty‐five weeks of carnitine supplementation (time × treatment × exercise interaction *p* = 0.5431; Figure 1b).

### 3.3 Whole‐body and skeletal muscle metabolism during exercise

**FIGURE 2:**
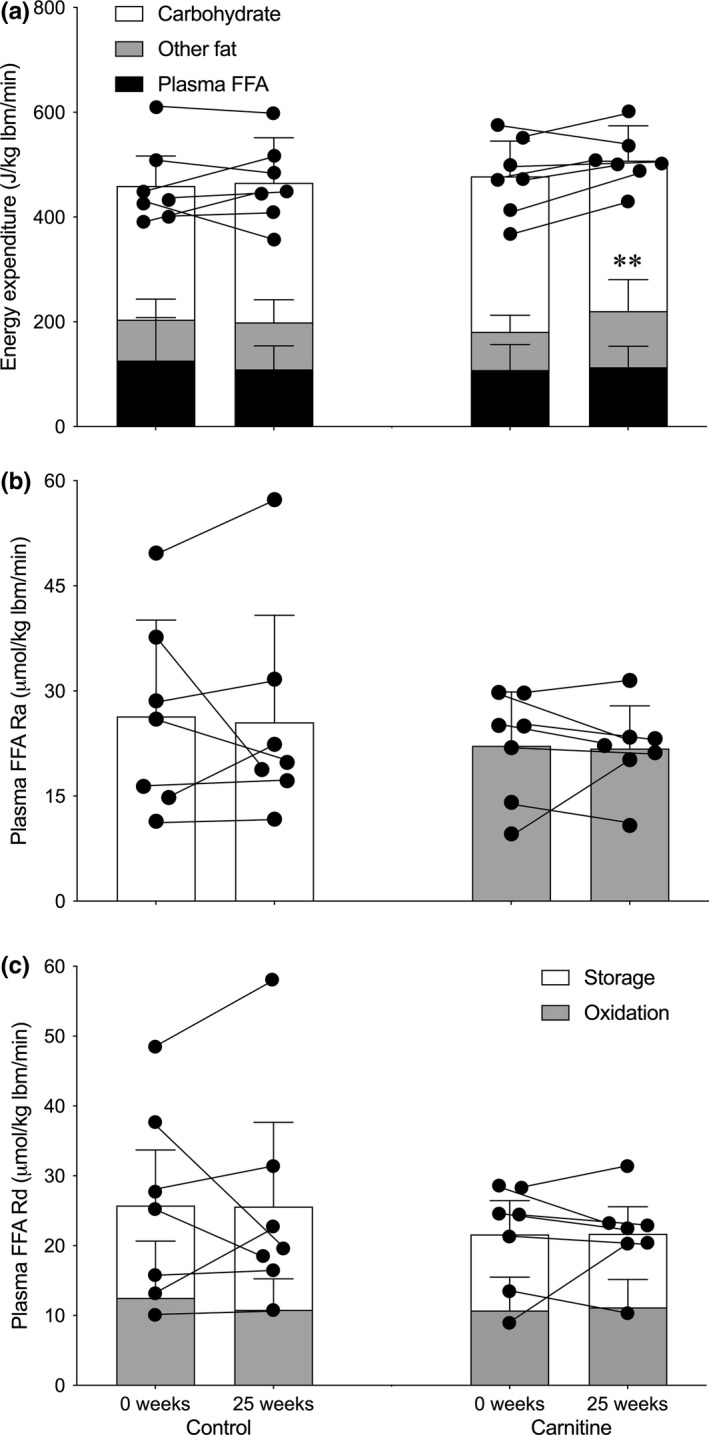
The contribution from the oxidation of plasma‐free fatty acids (FFA; filled bars), other fat sources (shaded bars), and carbohydrate (open bars) to total energy expenditure (a), the rate of appearance (Ra) of plasma FFA (b), and the proportion of plasma FFA rate of disappearance (Rd) during 1 h of exercise at 50% VO_2_max to storage and oxidation (c) before (0 weeks) and after 25 weeks of daily supplementation with 4.5 g of placebo (Control) or L‐carnitine L‐tartrate (Carnitine) in combination with 220 ml of a nutritional beverage containing 13.8 g protein and 44.4 g carbohydrate. Values represent mean ±SEM (*n* = 7). **p < 0.01, total fat oxidation at 25 weeks in Carnitine significantly different to corresponding 0 weeks value. Individual responses for total energy expenditure (a), plasma FFA Ra (b) and Rd (c) are represented by filled circles and connected with lines

Total energy expenditure (459.7 ± 39.5 to 465.4 ± 56.6 vs. 477.7 ± 71.2 to 507.8 ± 36.1 J/kg lean body mass (lbm)/min, respectively) and carbohydrate oxidation (256.4 ± 22.7 to 267.3 ± 29.9 vs. 296.5 ± 27.7 to 287.4 ± 27.3 J/kg lbm/min, respectively) were similar between Control and Carnitine before and after 25 weeks of supplementation when exercising at similar absolute workloads of 53.7 ± 15.2 and 48.4 ± 18.9 W, respectively (Figure 2a). In contrast, there was a time × treatment interaction for total fat oxidation (*p* < 0.05) such that it was increased by 21% during exercise from 181.1 ± 53.4 to 220.4 ± 67.3 J/kg lbm/min in Carnitine (*p* < 0.01). However, there was no difference in fatty acid oxidation from plasma or other sources (Figure 2a), plasma fatty acid Ra (Figure 2b), Rd (Figure 2c), or % of Rd oxidized (Figure 2c), or the change in IMF or SSL LD area, size or number during exercise between Control and Carnitine at baseline or after 25 weeks of supplementation (Table 2).

**TABLE 2:** Intramyofibrillar (IMF) and subsarcolemmal (SSL) lipid droplet (LD) content, size, and number before (Pre) and immediately after (Post) 1 h of exercise at 50% VO_2_max before (0 weeks) and after 25 weeks of daily supplementation with 4.5 g of placebo (Control) or L‐carnitine L‐tartrate (Carnitine) in combination with 220 ml of a nutritional beverage containing 13.8 g protein and 44.4 g carbohydrate

	Control				Carnitine			
	0 weeks		25 weeks		0 weeks		25 weeks	
	Pre	Post	Pre	Post	Pre	Post	Pre	Post
IMF LD content (%fibre area)*	0.79 ± 0.34	0.72 ± 0.51	0.87 ± 0.56	0.71 ± 0.42	0.61 ± 0.22	0.41 ± 0.15	0.94 ± 0.62	0.45 ± 0.23
IMF LD size (µm^2^)	0.31 ± 0.08	0.33 ± 0.14	0.29 ± 0.09	0.27 ± 0.09	0.31 ± 0.06	0.32 ± 0.09	0.42 ± 0.17	0.28 ± 0.05
IMF LD number (LD/mm^2^)*	24.8 ± 5.1	20.6 ± 7.3	30.0 ± 12.1	24.3 ± 9.9	23.2 ± 8.7	14.2 ± 4.7	28.6 ± 13.3	14.1 ± 5.4
SSL LD content (%fibre area)**	3.14 ± 2.34	3.31 ± 2.23	3.89 ± 2.52	2.22 ± 1.69	3.37 ± 2.65	4.09 ± 2.34	3.42 ± 2.34	2.53 ± 1.18
SSL LD size (µm^2^)**	0.31 ± 0.13	0.40 ± 0.20	0.31 ± 0.23	0.25 ± 0.13	0.36 ± 0.16	0.44 ± 0.16	0.30 ± 0.17	0.31 ± 0.14
SSL LD number (LD/mm^2^)	107.2 ± 68.6	91.7 ± 29.9	137.8 ± 60.1	80.7 ± 36.7	129.1 ± 127.4	132.8 ± 76.7	113.5 ± 48.2	93.2 ± 38.0

All values (*n* = 7) are means ±standard deviation (SD).

* *p* < 0.05, main effect of exercise.

** *p* < 0.05, time × exercise interaction.

**FIGURE 3:**
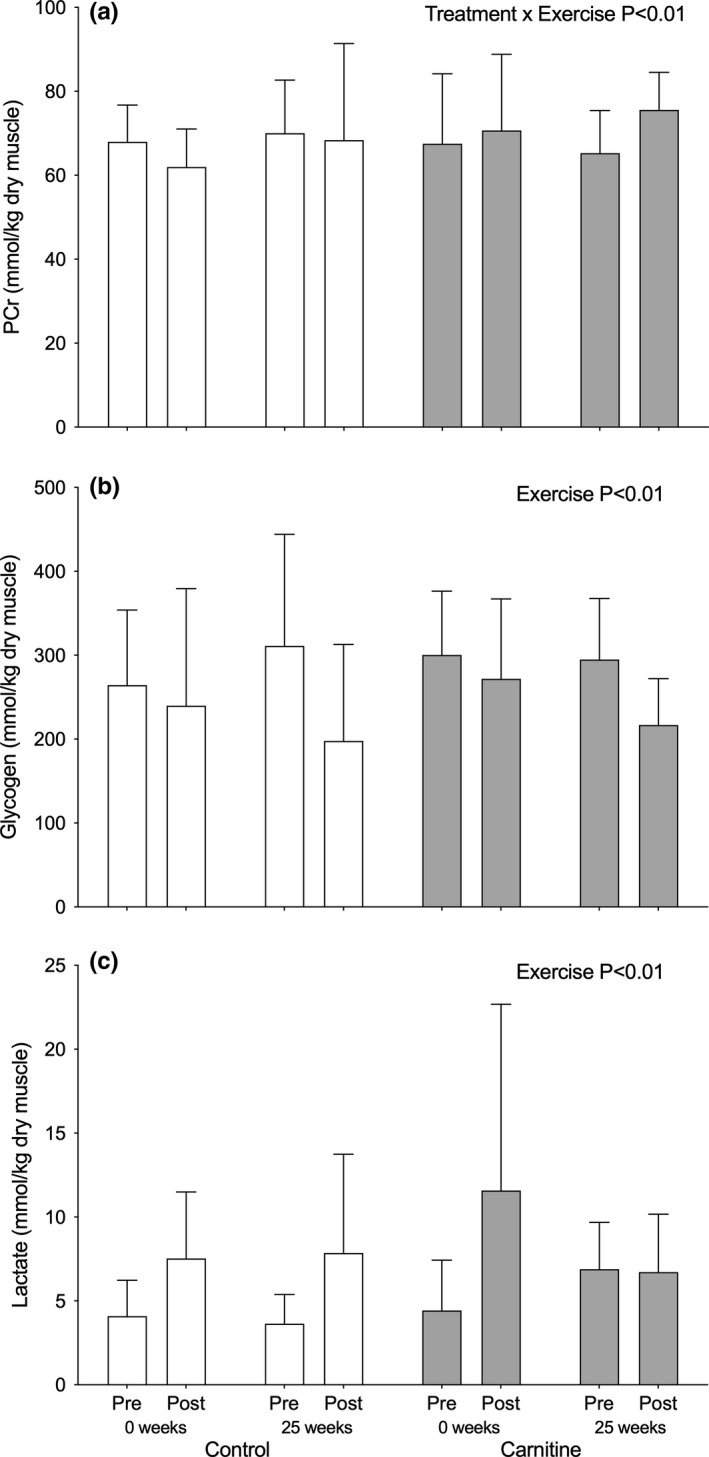
Skeletal muscle PCr (a), glycogen (b), and lactate (c) before (Pre) and immediately after (Post) 1 h of exercise at 50% VO_2_max after 25 weeks of daily supplementation with 4.5 g of placebo (Control; open bars) or L‐carnitine L‐tartrate (Carnitine; filled bars) in combination with 220 ml of a nutritional beverage containing 13.8 g protein and 44.4 g carbohydrate. Values represent mean ± SEM (*n* = 7). ^††^ *p* < 0.01, treatment × exercise effect, PCr increase during exercise significantly greater than corresponding Control

**FIGURE 4:**
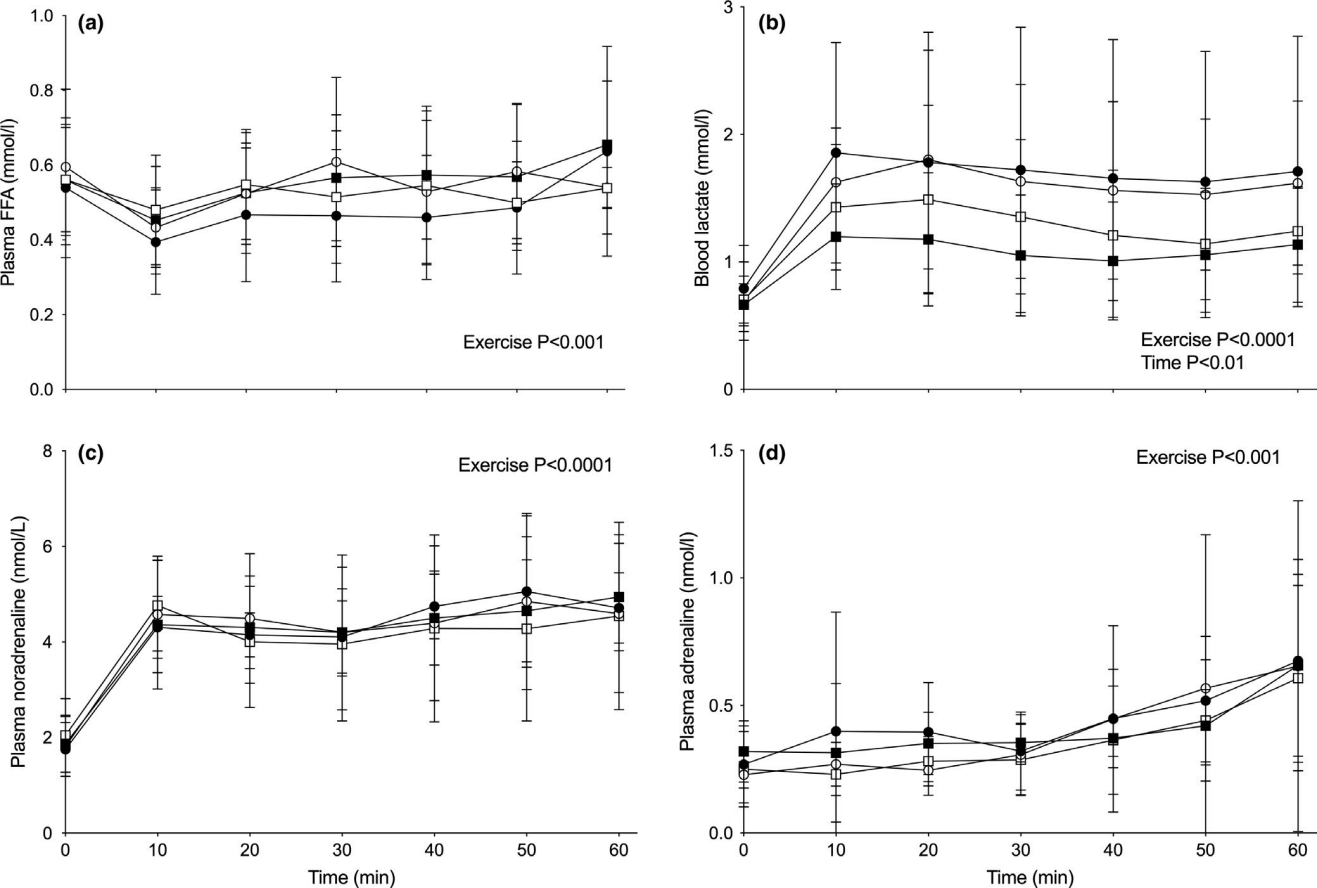
Plasma‐free fatty acid (FFA; a), blood lactate (b), and plasma noradrenaline (c) and adrenaline (d) concentration during 1 h of exercise at 50% VO_2_max before (circles) and after (squares) 25 weeks of daily supplementation with 4.5 g of placebo (Control; open symbols) or L‐carnitine L‐tartrate (Carnitine; filled symbols) in combination with 220 ml of a nutritional beverage containing 13.8 g protein and 44.4 g carbohydrate. Values represent mean ± SEM (*n* = 7)

There was a treatment × exercise interaction effect for skeletal muscle PCr content (*p* < 0.01; Figure 3a) and a main effect of exercise for skeletal muscle glycogen (*p* < 0.01; Figure 3b), lactate (*p* < 0.01; Figure 3c), carnitine (*p* < 0.0001; Figure 1b) and acetylcarnitine (*p* < 0.0001; Figure 1b). Thus, whereas PCr appeared to be reduced during exercise to a lesser degree in Carnitine compared to control before and after 25 weeks of supplementation, there was no difference between groups in the reduction in muscle glycogen and carnitine, and the reciprocal increases in muscle lactate and acetylcarnitine, following exercise. There was around a 0.7 mmol/l lower steady‐state blood lactate concentration during exercise in both Control and Carnitine following 25 weeks of supplementation (Figure 3b; *p* < 0.01, time x exercise interaction). However, there were no differences between groups in the increase in plasma fatty acid (Figure 3a; *p* < 0.01, exercise effect), noradrenaline (Figure 3c; *p* < 0.01, exercise effect) or adrenaline (Figure 3d; *p* < 0.01, exercise effect) concentrations during exercise, either before or after supplementation (Figure 4).

### 3.4 Insulin sensitivity and body composition

**FIGURE 5:**
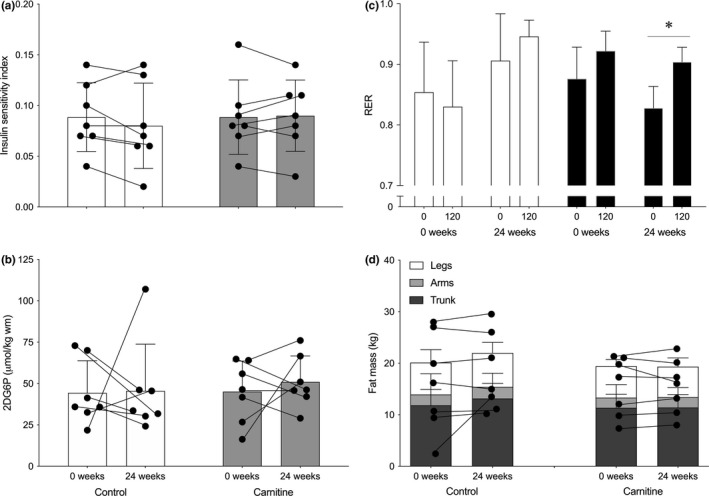
Steady‐state insulin sensitivity index (a), skeletal muscle 2‐deoxyglucose‐6‐phosphate accumulation (2DG6P; b), and fasting and 2 h insulin‐stimulated respiratory exchange ratio (RER; c) during a 3 hr hyperinsulinaemic (60 mU/m^2^/min) euglycaemic clamp, as well as legs (open bar), arms (shaded bar), and trunk (filled bar) fat mass measured by DXA (d), before (0 weeks; open bars, circles) and after (24 weeks; filled bars, squares) 24 weeks of daily supplementation with 4.5 g of placebo (Control; open symbols) or L‐carnitine L‐tartrate (Carnitine; filled symbols) in combination with 220 ml of a nutritional beverage containing 13.8 g protein and 44.4 g carbohydrate. Values represent mean ± SEM (*n* = 7). *p < 0.05, RER at 25 weeks in Carnitine significantly different to 0 weeks. Individual responses for insulin sensitivity index (a), muscle 2DG6P (b) and total fat mass (d) are represented by filled circles and connected with lines

The steady‐state rate of glucose disposal during the final hour of the 3‐h euglycaemic hyperinsulinaemic clamp was similar at 0 weeks between Control and Carnitine and did not change measurably after 24 weeks (48.3 ± 12.9 to 46.6 ± 17.1 vs. 51.2 ± 19.8 to 48.9 ± 15.5 μmol/kg lbm/min, respectively). Steady‐state serum insulin concentration was also similar between Control and Carnitine at 0 and 24 weeks (125.5 ± 18.0 to 134.6 ± 19.8 vs. 131.5 ± 17.6 to 127.7 ± 20.8 mIU/L, respectively). As such, there was no effect on IS_clamp_ between groups (Figure 5a). There was also no effect of 24 weeks of supplementation on skeletal muscle insulin sensitivity as measured by steady‐state plasma 2DG concentration between Control and Carnitine during the hyperinsulinaemic euglycaemic clamp (data not shown) and muscle 2DG6P content (Figure 5b; 44.3 ± 19.5 to 45.5 ± 28.3 vs. 45.1 ± 18.5 to 51.0 ± 15.7 μmol/kg wm respectively). However, there was a time x treatment interaction (*p* < 0.05) for RER, resulting in a lower RER in Carnitine before and during the hyperinsulinaemic euglycaemic clamp after 24 weeks (*p* < 0.05; Figure 5c). Whole‐body fat mass remained similar over 24 weeks of supplementation in both Control and Carnitine (20.1 ± 9.5 to 22.0 ± 7.5 vs. 19.4 ± 5.7 to 19.3 ± 5.2 kg; Figure 5d). Indeed, there was no difference in trunk, arm, or leg fat mass within or between Control and Carnitine over the course of the study (Figure 5d).

### 3.5 Skeletal muscle gene expression

The fold change in expression of skeletal muscle transcripts involved in fatty oxidation (ACAT1) and IMCL turnover (DGKD and PLIN2) was greater (*p* < 0.05) in Carnitine compared with Control at 24 weeks (Table 3), whereas changes in other measured transcripts were similar between groups.

**TABLE 3:** Fold change in expression of skeletal muscle transcripts encoding proteins involved in fatty acid oxidation and IMCL turnover following 24 weeks of daily supplementation with 4.5 g of placebo (Control) or L‐carnitine L‐tartrate (Carnitine) in combination with 220 ml of a nutritional supplement containing 13.8 g protein and 44.4 g carbohydrate

	Gene	Control	Carnitine
Fatty acid oxidation	ACACB	1.19 ± 0.59	1.32 ± 0.71
	CPT1B	1.95 ± 2.02	1.17 ± 0.58
	CPT2	1.42 ± 0.61	0.99 ± 0.44
	HADHB	0.80 ± 0.80	1.09 ± 0.46
	ACADM	0.78 ± 0.43	1.08 ± 0.41
	ACAT1	0.82 ± 0.51	1.33 ± 0.31*
IMCL turnover	SPTLC1	1.17 ± 0.83	1.15 ± 0.36
	DGKD	0.85 ± 0.11	1.23 ± 0.17^+^
	DGAT1	0.93 ± 0.31	1.05 ± 0.16
	PLIN2	0.76 ± 0.56	1.23 ± 0.23^+^
	PLIN5	1.26 ± 0.81	1.48 ± 1.39
	PNPLA2	1.82 ± 0.93	1.25 ± 0.54

All values (*n* = 7) are means ±standard deviation (SD).

* *p* < 0.05, Carnitine different to corresponding Control value.

## 4 DISCUSSION

The aim of the present study was to test the hypothesis that a dietary mediated increase in skeletal muscle carnitine content would increase the rate oxidation of plasma fatty acid during moderate‐intensity exercise in older male participants. We also aimed to assess whether any effect would be of a magnitude to reduce resting IMCL content, insulin resistance and fat mass over 25 weeks of moderate‐intensity exercise prescription in older individuals. Thus, the main finding from the present study was that a 20% increase in muscle total carnitine content resulted in around a 20% increase in whole‐body total fatty acid oxidation during exercise at 50% VO_2_max, which was likely due to an increase in IMCL, rather than plasma fatty acid, utilization. However, this apparent increase in IMCL turnover during moderate‐intensity exercise did not translate into a reduction in resting SSL IMCL content or an improvement in insulin sensitivity and whole‐body and regional fat mass. Whether this was because the exercise prescription volume and/or duration was not of sufficient magnitude to translate the increased fat oxidation into a measurable change in IMCL content, requires further investigation, but it would appear that a carnitine‐mediated increase in mitochondrial fatty acid flux is achievable in older individuals.

We have previously demonstrated that twice daily ingestion of 1.36 g L‐carnitine for 12 to 24 weeks, in combination with a beverage containing 80 g of carbohydrate in order to stimulate insulin‐mediated muscle carnitine accumulation, can increase skeletal muscle total carnitine content by around 20% in young healthy men (Stephens et al., 2013; Wall et al., 2011). This was in line with our previous research demonstrating that intravenously infusing L‐carnitine in the presence of high circulating insulin (>50 mU/L) increased muscle carnitine content by 15% (Stephens et al., 2006a, 2007), and that ingesting >80 g carbohydrate in a beverage increased whole‐body (Stephens et al., 2007) and forearm muscle (Shannon et al., 2016) carnitine retention when combined with 3 g of L‐carnitine feeding. A novel finding of the present study was that combining 3 g/day supplementation of L‐carnitine with a protein containing insulinogenic beverage with lower carbohydrate content (44.4 vs. 80 g), known to increase serum insulin concentration to around 100 mU/L (Dirks et al., 2020), increased skeletal muscle total carnitine content by around 20% in older individuals (~20 to ~24 mmol/kg dm). A recent study by Bruls et al. (2019) demonstrated that ingestion of 1.36 g/d L‐carnitine (2 g L‐carnitine L‐tartrate) for just 36 days could increase muscle total carnitine content by around 12% (8.5–9.5 mmol/kg dm) in 60‐year‐old individuals with impaired glucose tolerance. This increase is in line with the present findings, as well as previous calculations that ingestion of 3 g of L‐carnitine combined with an insulinogenic beverage would increase the muscle store by 0.1–0.3%/day (Shannon et al., 2016; Stephens, Evans, et al., 2007). The fact that an insulinogenic beverage was not used in the study of Bruls et al. (2019) is difficult to reconcile with previous findings that neither daily feeding of L‐carnitine *per se* for up 3 months (Wächter et al., 2002), nor intravenously infusing L‐carnitine for up to 5 h (Stephens et al., 2006a), had an effect on muscle total carnitine content, or indeed net uptake of carnitine across the leg (Soop et al., 1988) or forearm (Shannon et al., 2016) in the absence of insulin. However, possible explanations could be that the L‐carnitine dose was spilt across three meals that were presumably very insulinogenic in a cohort with impaired glucose tolerance and able to stimulate muscle carnitine uptake, or that the individuals with impaired glucose tolerance were muscle carnitine deficient (8.5 mmol/kg dm).

A major finding of the present study was that the increase in muscle carnitine content was associated with a remarkable 20% increase in the rate of whole‐body total fat oxidation during exercise at 50% VO_2_max, compared with placebo group response that was unchanged. The pre‐supplementation values for whole‐body total fat oxidation during exercise, as well as those of total energy expenditure and the relative contribution of plasma and other fat sources, were directly in line with previous studies in older individuals exercising at the same intensity (Boon et al., 2007). The post‐supplementation values are also in line with our previously observed carnitine‐mediated 10% increase in fatty acid oxidation and fourfold increase in muscle (presumably mitochondrial) long‐chain acyl‐CoA content in younger individuals (Stephens et al., 2013). As several studies have demonstrated reduced fat oxidation in older individuals despite increased whole‐body lipolysis and circulating fatty acid availability compared to young at rest and during exercise (Boon et al., 2007; Chee et al., 2016; Sial et al., 1996; Solomon et al., 2008), we hypothesized that increasing muscle carnitine availability would increase circulating fatty acid oxidation. However, by using an intravenous [U‐^13^C]palmitate infusion approach combined with electron microscopy analysis of net IMCL utilization, we demonstrated no change in plasma fatty acid oxidation with increased whole‐body total fat oxidation, and no change in plasma fatty acid rate of appearance (presumably from adipose tissue and consistent with the unaltered catecholamine concentrations), rate of disappearance (half of which is thought to enter exercising skeletal muscle), or the proportion of fatty acids leaving the circulation that are oxidized rather than stored. On the other hand, we did observe a time x exercise interaction effect for SSL IMCL content, which may suggest that IMCL utilization drove the increase in fat oxidation in Carnitine despite SSL IMCL content also decreasing in Control. The reason for a selective oxidation of IMCL over plasma fatty acids is unclear but is perhaps due to an exercise training effect combined with a carnitine‐mediated increase in energy expenditure, particularly given the rate of fatty acid disappearance did not change. Indeed, we have previously shown that an 80% increase in muscle‐free carnitine availability during exercise at 50% VO_2_max in younger individuals resulted in a 6% increase in energy expenditure (Stephens et al., 2013). However, while there was a numerical 6% increase in energy expenditure during exercise in the present study, it did not reach statistical significance (time × exercise interaction *p* = 0.340). Alternatively, the selective oxidation of IMCL could also be due to a local increase of free carnitine content in the vicinity of IMF mitochondrial IMCL (i.e. lipid droplets proximal to mitochondria) as we have previously speculated (van Loon et al., 2001; Stephens, Constantin‐Teodosiu, et al., 2007; Stephens & Galloway, 2013), particularly given free carnitine availability increased by 50% during exercise.

The premise of the present study was that a carnitine‐mediated increase in total fat oxidation over 25 weeks of moderate‐intensity exercise prescription would lead to a reduction in IMCL content. We have previously demonstrated that only IMF, but not SSL, IMCL content declines with exercise in lean active older individuals, predominantly due to a reduction in lipid droplet number, and that SSL IMCL content increases with exercise in overweight sedentary older individuals, predominantly due to increased lipid droplet size (Chee et al., 2016). We observed a similar pattern before exercise prescription in the present study, but an increase in IMF IMCL utilization and a reduction in SSL IMCL accumulation during exercise after training regardless of treatment. However, this did not result in a decline in IMCL content after 25 weeks of supplementation, even when total fat oxidation was increased with muscle carnitine loading. The few pertinent exercise training studies of older individuals (~67 y) demonstrated that 12 weeks of exercise training (30–60 min at 60–75% VO_2_max, 4–6 times/week) increased basal fat oxidation (Solomon et al., 2008) and mitochondrial content and function (Menshikova et al., 2006; Pruchnic et al., 2004). Moreover, exercise training at similar intensities has been shown to improve basal fat oxidation and prevent IMCL accumulation (Sial et al., 1996; Solomon et al., 2008), particularly in the SSL region (Li et al., 2014; Nielsen et al., 2010). Notably, these studies involved substantially greater training intensities / frequencies over a shorter period of time than were used in our study, which would suggest that a carnitine‐mediated increase in fat oxidation may be required in combination with a larger training load, or that muscle carnitine content should be elevated in the months before prolonged L‐carnitine supplementation and training commences. Indeed, we have previously demonstrated that carnitine supplementation in younger trained individuals who were partaking in regular high‐intensity endurance exercise increased skeletal gene muscle expression of the PNPLA2, which encodes for adipose triglyceride lipase the rate‐limiting step in IMCL hydrolysis, and PNPLA2 gene expression was unchanged with carnitine loading in the present study. Nevertheless, the expression of other genes involved in IMCL turnover, PLIN2 (perilipin 2), and lipid intermediate metabolism, DGKD (diacylglycerol kinase delta), was increased with carnitine supplementation, and it is generally accepted that the accumulation of metabolites associated with IMCL (i.e. acyl‐CoA, diacylglycerol and ceramide) is a major cause of insulin resistance in skeletal muscle with age (Lee et al., 2010; Savage et al., 2007).

Despite a trend for a decrease in fasting blood glucose in Carnitine after 25 weeks, a lack of an effect of increased skeletal muscle carnitine content *per se* on insulin sensitivity was somewhat surprising. We have previously demonstrated that a 15% increase in skeletal muscle total carnitine, achieved via intravenous L‐carnitine infusion during a 6 h euglycaemic hyperinsulinaemic clamp in healthy young volunteers at rest, increased muscle glycogen content by 30% the following morning under controlled dietary conditions (Stephens et al., 2006b). Indeed, acute intravenous carnitine administration in combination with a euglycaemic hyperinsulinaemic clamp has been previously demonstrated to improve whole‐body glucose disposal in normal healthy participants and individuals with type 2 diabetes (Capaldo et al., 1991; De Gaetano et al., 1999; Giancaterini et al., 2000; Mingrone et al., 1999). Incomplete or insufficient β‐oxidation of fatty acids, reflected by acylcarnitine accumulation, has been implicated in the development of skeletal muscle insulin resistance (Koves et al., 2008), and there is growing interest in the role of skeletal muscle carnitine and its associated enzymes in buffering these negative effects. For example, Bruls et al., (2019) observed that increasing skeletal muscle carnitine content by 12% in individuals with impaired glucose tolerance increased basal fat oxidation by around 10% and improved metabolic flexibility during a mixed meal tolerance test. Although we did not observe any increase in metabolic flexibility during the insulin clamp, nor an increased muscle acetylcarnitine content (i.e. increased acetyl‐CoA buffering) during exercise with elevated free carnitine availability, we did observe an increase in fat oxidation under basal and insulin‐stimulated conditions following carnitine loading. This is consistent with the increase in fat oxidation during exercise, and was associated with an increase in gene expression of ACAT1, the gene encoding acetyl‐CoA acetyltransferase (also known as acetoacetyl‐CoA thiolase), which we have previously demonstrated to be a main effect of muscle carnitine loading (Stephens et al., 2013). Acetoacetyl‐CoA thiolase is abundant in skeletal muscle where it functions both in ketogenesis and ketolysis (from both fatty acids and amino acids), and its maximal activity has been demonstrated to increase with exercise training (Winder et al., 1975). We have also previously shown that ACAT1 expression was reduced in older individuals, which was associated with insulin resistance and a blunted stimulation of IMCL turnover in response to exercise (Chee et al., 2016; Tsintzas et al., 2017). Whether this increase in ACAT1 is a cause or effect of increased mitochondrial fatty acid flux requires further investigation, but it perhaps provides another mechanism whereby increased acetyl‐CoA production from excessive mitochondrial substrate flux can be buffered.

In conclusion, we show for the first time that increasing skeletal muscle total carnitine content by 20% in older individuals resulted in an increase in whole‐body total fatty acid oxidation at rest and during exercise at 50% VO_2_max. The increase in whole‐body total fat oxidation did not translate into reduced IMCL content, an improved ability to acetylate‐free carnitine or improved insulin sensitivity. It also did not affect body composition, markers of physical or mental function, or ability to perform activities of daily living. These latter findings should be taken with some caution given the low number of participants in the present study and chance of a type 2 error in some of the variables. However, given that a carnitine‐mediated increase in mitochondrial fatty acid flux is achievable in older individuals during moderate‐intensity exercise and that reduced lipid turnover is associated with poor metabolic health in older people, further exploration of alternative carnitine loading and exercise strategies are warranted.

## CONFLICTS OF INTEREST

The authors have no potential conflicts of interest to declare.

## AUTHOR CONTRIBUTIONS

The experiments in this paper were conducted in the School of Life Sciences, University of Nottingham. FBS, PLG and KS conceived and designed the work. CC, CES, AB, ALS, DW and FBS performed acquisition, analysis and interpretation of data. CC and FBS drafted the manuscript. All authors approved the final version of the manuscript to be published, and all authors contributed to drafting the article and revising it critically for important intellectual content. All authors agree to be accountable for all aspects of the work in ensuring that questions related to the accuracy or integrity of any part of the work are appropriately investigated and resolved. All persons designated as authors qualify for authorship, and all those who qualify for authorship are listed.

## Data Availability

The data that support the findings of this study are available on request from the corresponding author. The data are not publicly available due to privacy or ethical restrictions.
